# Therapeutical Notes

**Published:** 1899-01-07

**Authors:** 


### THERAPEUTICAL NOTES.

### Articular Rheumatism and Gout.

Friedrich Kölbl (*Wien Medicin. Zeitung* xliii., No. 5 and 6) recommends the application of bandages soaked in a solution as follows:—

Icthyol, 50 parts.
Glycerin, 20 parts.
Water, 30 parts.

This dressing is placed on the painful joints, covered with an impervious fabric, and kept hot for some hours. A bag of hot sand may be laid outside, and the application may be employed every evening.

